# Ivermectin as a SARS-CoV-2 Pre-Exposure Prophylaxis Method in Healthcare Workers: A Propensity Score-Matched Retrospective Cohort Study

**DOI:** 10.7759/cureus.17455

**Published:** 2021-08-26

**Authors:** Jose Morgenstern, Jose N Redondo, Alvaro Olavarria, Isis Rondon, Santiago Roca, Albida De Leon, Juan Canela, Johnny Tavares, Miguelina Minaya, Oscar Lopez, Ana Castillo, Ana Placido, Rafael Cruz, Yudelka Merette, Marlenin Toribio, Juan Francisco

**Affiliations:** 1 Infectious Diseases, Centro Medico Punta Cana, Punta Cana, DOM; 2 Cardiology, Grupo Rescue, Puerto Plata, DOM; 3 Data Analysis, Grupo Rescue, Santiago, CHL; 4 Nursing, Grupo Rescue, Puerto Plata, DOM; 5 Quality Director, Grupo Rescue, Punta Cana, DOM; 6 Anesthesiology, Centro Medico Punta Cana, Punta Cana, DOM; 7 Obstetrics and Gynecology, Centro Medico Canela, La Romana, DOM; 8 Pulmonology, Grupo Rescue, Puerto Plata, DOM; 9 Internal Medicine, Centro Medico Punta Cana, Punta Cana, DOM; 10 Emergency Department, Centro Medico Bournigal, Puerto Plata, DOM; 11 Emergency Department, Centro Medico Punta Cana, Punta Cana, DOM; 12 Critical Care, Centro Medico Bournigal, Puerto Plata, DOM; 13 Critical Care, Centro Medico Punta Cana, Punta Cana, DOM

**Keywords:** sars-cov-2, covid-19, pre-exposure prophylaxis, prep, healthcare worker, ivermectin, coronavirus, covid-19 prevention

## Abstract

Background: Ivermectin is a drug that has been shown to be active against coronavirus disease 19 (COVID-19) in previous studies. Healthcare personnel are highly exposed to severe acute respiratory syndrome coronavirus 2 (SARS-CoV-2) infection. Therefore, we decided to offer them ivermectin as a pre-exposure prophylaxis (PrEP) method.

Purpose: Primary outcome was to measure the number of healthcare workers with symptomatic SARS-CoV-2 infection and a positive reverse transcription polymerase chain reaction (RT-PCR) COVID-19 test in the ivermectin group and in the control group. Secondary outcome was to measure the number of sick healthcare workers with a positive RT-PCR COVID-19 test whose condition deteriorated and required hospitalization and/or an Intensive Care Unit (ICU), or who died, in the ivermectin group and in the control group.

Material and methods: This observational and retrospective cohort study was carried out in two medical centers, Centro Medico Bournigal (CMBO) in Puerto Plata and Centro Medico Punta Cana (CMPC) in Punta Cana, Dominican Republic. The study began on June 29, 2020, and ended on July 26, 2020. A Statistical Package for Social Sciences (SPSS) Propensity Score Matching procedure was applied in a 1:1 ratio to homogeneously evaluate 271 healthcare personnel that adhered to a PrEP program with ivermectin at a weekly oral (PO) dose of 0.2 mg/kg, and 271 healthcare personnel who did not adhere to the program were assigned as a control group.

Results: In 28 days of follow-up, significant protection of ivermectin preventing the infection from SARS-CoV-2 was observed: 1.8% compared to those who did not take it (6.6%; p-value = 0.006), with a risk reduction of 74% (HR 0.26, 95% CI [0.10,0.71]).

Conclusions: These results suggest that compassionate use of weekly ivermectin could be an option as a preventive method in healthcare workers and as an adjunct to immunizations, while further well-designed randomized controlled trials are developed to facilitate scientific consensus.

## Introduction

Ivermectin, commonly used as an antiparasitic, has shown not only antiviral activity against the severe acute respiratory syndrome coronavirus 2 (SARS-CoV-2) in-vitro [1], but there are already studies of its viricidal action in humans [2-3]. Peer-reviewed systematic reviews and meta-analyses also show a prophylactic effect [4-5]. The Grupo Rescue added its own pioneering experience in the use of ivermectin in the early treatment of coronavirus disease 2019 (COVID-19)-infected patients in the Dominican Republic at the beginning of the pandemic [6].

Healthcare personnel are highly exposed to SARS-CoV-2 infection. Therefore, it would be very wise and convenient to have a pre-exposure prophylaxis (PrEP) oral medication at hand.

At a time of a wave of contagion with COVID-19 in the Dominican Republic, with several seriously infected in our healthcare personnel, we were forced to start with ivermectin as a PrEP method to try to reduce the incidence of the SARS-CoV-2 infection. This moved us several months later to collect the data in order to perform a retrospective observational cohort study and evaluate the results. The primary outcome measure of this study was the number of participants with symptomatic SARS-CoV-2 infection and a positive COVID-19 reverse transcription polymerase chain reaction (RT-PCR) test in the ivermectin group and in the control group. The secondary outcome was the number of sick participants with a positive RT-PCR COVID-19 test, whose condition deteriorated and required hospitalization and/or an Intensive Care Unit (ICU) and the number of sick participants with a positive RT-PCR COVID-19 test who died in the ivermectin group and in the control group.

This article was previously posted to the medRxiv preprint server on 04/17/2021. Last version: 06/04/2021.

## Materials and methods

This study is an observational retrospective multicenter cohort conducted on active healthcare workers at the Centro Medico Bournigal (CMBO) and the Centro Medico Punta Cana (CMPC), Dominican Republic, following the suggested guidelines of the Strengthening the Reporting of Observational Studies in Epidemiology (STROBE) statement for reporting observational studies [7]. The study protocol is available at Clinicaltrials.gov, identifier NCT04832945.

This study started on June 29, 2020, and ended on July 26, 2020, followed up for 28 days, in patients who were invited to a voluntary SARS-CoV-2 PrEP compassionate program using ivermectin at a weekly PO dose of 0.2 mg/kg, administered at the Human Resources Office. They were compared with a control group of healthcare personnel who did not receive ivermectin, either by their own will or because of contraindications in its use. Before the PrEP started it was submitted for consideration, comment, guidance and approval to the Grupo Rescue Ethics Committee who issued approval ME-GRUR-328-2020.

Both the ivermectin group and the control group strictly followed the preventive measures and the use of personal protection equipment (PPE) to avoid SARS-CoV-2 infection, implemented since April 2020, well before the start of this study. The SARS-CoV-2 symptomatic infected cases were confirmed by an RT-PCR COVID-19 test.

The participants who adhered to the ivermectin prophylaxis program had signed the informed consent, were a minimum age of 18 years old, and were included in the intervention group only if: 1. received the first dose of ivermectin during the first week of the study; 2. complied with a minimum of two doses of ivermectin during the four weeks of the study or one dose if RCT-PCR positive within 14 days of intake; 3. the difference in days between the two doses was no greater than 14 days. The participants had weekly presentations in the Human Resources Office, where the medication was orally administered, being recorded the date of the ivermectin delivery.

The following healthcare personnel were excluded from the ivermectin prophylaxis program: pregnant or suspected pregnant women, women breastfeeding, patients receiving coumarin anticoagulants, and those allergic to ivermectin.

The healthcare personnel who did not adhere to the program until July 26 were assigned to the control group and at no time before or during the study did they take ivermectin.

Those who had a positive RT-PCR COVID-19 test previous to the start of the study were excluded.

To obtain the demographic data of the workers, they were extracted from the Sigma software in CMPC and an internal development software in CMBO, from which the worker's code, date of birth, sex and role or position were obtained. With this information, the Head of the Nursing Department assigned an exposure level, which was defined as follows: 1. High: personnel in direct contact with COVID-19 patients; 2. Medium: personnel who care for non-COVID-19 patients; 3. Low: personnel who do not take care of patients (administrative, others).

The compilation of information from RT-PCR COVID-19 reports was done by consulting the Referencia Laboratorio Clínico system, located in Santo Domingo, Dominican Republic, who performed the analysis of the nasopharyngeal samples from both centers. Additionally, the healthcare personnel who gave an RT-PCR COVID-19 (+) during the study were registered in their respective files in the Human Resources Office. The records of both medical centers were consolidated into a single record by the Head of the Nursing Department, individualizing the worker, date of report and outcome (hospitalization and/or death).

Those participants in the study who tested positive for COVID-19 were treated as outpatients and followed by their respective medical center with ivermectin 0.4 mg/Kg PO one dose and azithromycin 500 mg PO every 24 hours for five days. Those who required hospitalization received ivermectin 0.3 mg/kg PO on days one, two, six, and seven plus azithromycin 500 mg PO every 24 hours for seven days.

Statistical analysis

To determine the degree of balance of the cohorts and to adjust the possible confounders of the study, a chi-square homogeneity test was performed. Those variables that obtained a p-value < 0.1 test were included in the Propensity Score Matching (PSM) process of the Statistical Package for Social Sciences (SPSS; IBM Corp., Armonk, NY, USA) software, in which a 1:1 match was performed with a tolerance of 0.05 to obtain homogeneous cohorts. These variables were gender, exposure and role.

After performing the matching, a chi-square test and a Kaplan-Meier hazard curve were performed with the positive COVID-19 test results of the healthcare personnel. Thereafter a Cox regression was performed to compare the effect of the study variables on the primary outcome, which was a positive COVID-19 test. The validation and creation of the cohorts were done in MariaDB 10 (mariadb.org). The statistical analysis was done with SPSS and RStudio 1.4 (R Foundation for Statistical Computing, Vienna, Austria).

## Results

Initially 943 candidates were selected for the study, from which 43 were excluded for having a positive COVID-19 RT-PCR report before the start of the study. The ivermectin group was made up of 510 healthcare personnel and the control group was made up of 390 participants. In total, 182 healthcare workers who did not comply with the ivermectin doses described in the inclusion criteria were excluded from the ivermectin group. In the follow-up study, five participants were lost who resigned or were dismissed from their work, two in the ivermectin group and three in the control group. This equates to a 0.6% loss of the follow-up for the ivermectin group and 0.77% for the control group. As it is less than 5%, it is not considered relevant for the purposes of the study.

**Figure 1:**
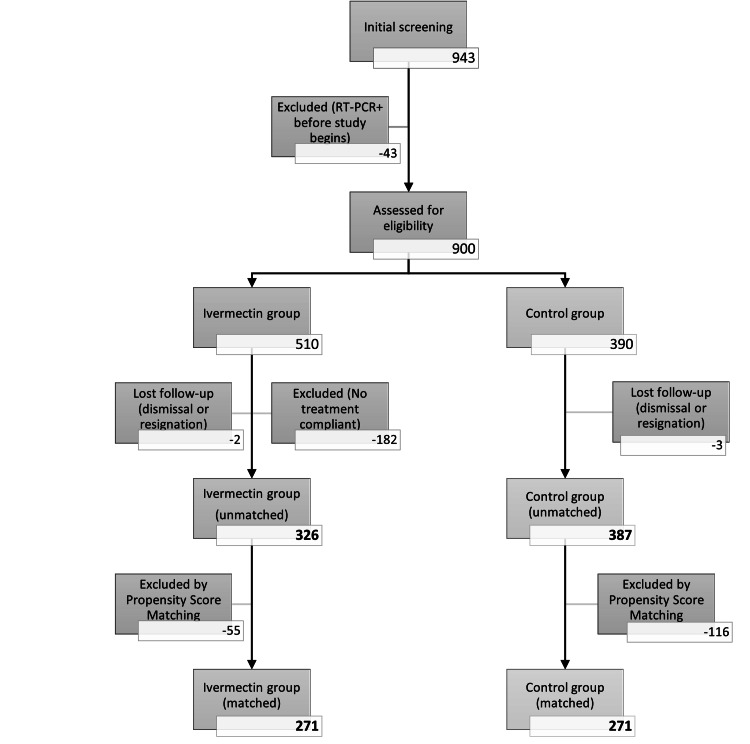
Evolution of the cohorts throughout the study flowchart

Finally, 713 participants were chosen, 326 in the ivermectin group and 387 in the control group, and were considered for the preliminary analysis of this study. After the matching process with the Propensity Score, there were 271 members in each group for analysis (Figure 1).

51.5% of the participants in the ivermectin group took four doses, 40.4% took three doses, 6.7% took two doses, and 1.4% of the participants managed to take a single dose and then reported a positive COVID-19 infection. The median time between doses was seven days and the mean (average) was 7.2 days with a standard deviation of 1.03.

There were no severe side effects reported from the use of ivermectin, only minor side effects such as dizziness (3.7%), headache (1.5%), stomachache (1.4%), pruritus (1.1%), nausea (1.1%) and diarrhea (0.7%).

Demographic data

Executing the Propensity Score Matching process using the gender, exposure and role variables, the groups were left without significant differences in the measured variables (Table 1).

**Table 1:** Chi-square homogeneity test of demographic characteristics of the participants at the beginning of the study. Unmatched and matched groups comparison. CMBO: Centro Medico Bournigal, CMPC: Centro Medico Punta Cana

	Unmatched cohort				Matched cohort			
Characteristic	IVM (n=326)	Control (n=387)	Total (n=713)	p-value	IVM (n=271)	Control (n=271)	Total (n=542)	p-value
Age	35.14 ± 9.44	35.71 ± 10.72	35.45 ± 10.15	0.456	35.21 ± 9.33	35.14 ± 10.67	35.19 ± 10.02	0.928
Age group								
< 30 years	105 (32.2)	125 (32.3)	230 (32.3)	0.663	86 (31.7)	93 (34.3)	179 (33.0)	0.894
30-39 years	126 (38.7)	147 (38.0)	273 (38.3)		106 (39.1)	98 (36.2)	204 (37.6)	
40-49 years	68 (20.9)	73 (18.9)	141 (19.8)		56 (20.7)	56 (20.7)	112 (20.7)	
> 50 years	27 (8.3)	42 (10.9)	69 (9.7)		23 (8.5)	24 (8.9)	47 (8.7)	
Gender								
Male	56 (17.2)	87 (22.5)	147 (20.6)	0.078	56 (20.7)	58 (21.4)	114 (21.0)	0.833
Female	270 (82.8)	300 (77.5)	577 (80.9)		215 (79.3)	213 (78.6)	428 (79.0)	
Location								
CMBO	177 (54.3)	193 (49.9)	370 (51.9)	0.239	142 (52.4)	130 (48.0)	272 (50.2)	0.303
CMPC	149 (45.7)	194 (50.1)	343 (48.1)		129 (47.6)	141 (52.0)	270 (49.8)	
Role								
Physician	27 (8.3)	95 (24.5)	122 (17.1)	< 0.001	27 (10.0)	29 (10.7)	56 (10.3)	0.965
Nurse	71 (21.8)	106 (27.4)	177 (24.8)		71 (26.2)	69 (25.5)	140 (25.8)	
Assistant	33 (10.1)	39 (10.1)	72 (10.1)		33 (12.2)	30 (11.1)	63 (11.6)	
Other	195 (59.8)	147 (38.0)	342 (48.0)		140 (51.7)	143 (52.8)	283 (52.2)	
Exposure Level								
High	83 (25.5)	155 (40.1)	238 (33.4)	< 0.001	82 (30.3)	80 (29.5)	162 (29.9)	0.759
Medium	84 (25.8)	85 (22.0)	169 (23.7)		71 (26.2)	65 (24.0)	136 (25.1)	
Low	159 (48.8)	147 (38.0)	306 (42.9)		118 (43.5)	126 (46.5)	244 (45.0)	

The mean age of the study was 35.19 years (±10.02), composed mainly of females (79.0%). Regarding the profession or role, physicians represent 10.3%, nurses 25.8%, medical assistants 11.6% and administrative personnel and others 52.2%. More than half of the personnel had medium and high exposure to COVID-19, corresponding to 29.9% and 25.1% (Table 1).

Comorbidities were not included in the demographic data as the information was not complete in 8.1%. However, with the existing data, this variable did not represent an imbalance between the ivermectin group and the control group (chi-square homogeneity test p-value=0.890) and in the Cox regression it also did not show to be a variable that affects the main outcome (HR 0.97, 95% CI [0.31, 3.08], p-value=0.962).

Primary and secondary outcomes

The chi-square homogeneity test showed that PrEP with ivermectin was associated with a statistically significant reduction in SARS-CoV-2 infection, with 1.8% versus 6.6% in the control group (p-value=0.006).

No progression of COVID-19 disease or complications which deserved hospital admission were observed and there were no mortalities in the ivermectin group. In the control group 0.7% showed clinical complications that warranted hospitalization, but there were no deaths. When comparing both groups the difference was not statistically significant in deterioration that required hospitalization and/or ICU (p-value=0.499) and in death (p-value=1.000) (Table 2).

**Table 2:** Chi-square homogeneity test outcomes 95% CI. Comparison of unmatched and matched groups. Fisher exact test for deterioration outcome p-value. IVM: ivermectin

	Unmatched cohort				Matched cohort			
Outcomes	IVM (n=326)	Control (n=387)	Total (n=713)	p-value	IVM (n=271)	Control (n=271)	Total (n=542)	p-value
Covid+	6 (1.8)	22 (5.7)	28 (3.9)	0.008	5 (1.8)	18 (6.6)	23 (4.2)	0.006
Deterioration	0 (0.0)	3 (0.8)	3 (0.4)	0.254	0 (0.0)	2 (0.7)	2 (0.4)	0.499
Death	0 (0.0)	0 (0.0)	0 (0.0)	1.000	0 (0.0)	0 (0.0)	0 (0.0)	1.000

The Cox regression analysis showed that ivermectin reduced the risk of COVID-19 infection by 74% compared to the control group (HR 0.26, 95% Cl [0.10, 0.71]). None of the analyzed covariables like age, sex, workplace location (CMBO or CMPC), occupation, or degree of exposure to COVID-19 significantly reduced or increased the risk of developing a COVID-19 infection (Table 3).

**Table 3:** Multivariate analysis (Cox regression) on COVID-19 infection outcome, 95% CI. Unmatched and matched groups comparison. CMBO: Centro Medico Bournigal

	Unmatched Cohort				Matched Cohort			
	95% CI for HR				95% CI for HR			
Variables	Hazard ratio	Lower	Upper	p-value	Hazard ratio	Lower	Upper	p-value
Age	1.00	0.97	1.04	0.879	0.99	0.94	1.03	0.552
Gender (Male)	1.63	0.65	4.10	0.298	1.12	0.37	3.36	0.837
Location (CMBO)	1.61	0.75	3.49	0.224	1.38	0.59	3.24	0.453
Role (Physician)	0.74	0.18	3.06	0.681	1.22	0.26	5.67	0.804
Role (Nurse)	1.27	0.39	4.18	0.676	0.92	0.26	3.37	0.906
Role (Assistant)	0.44	0.07	2.74	0.377	0.42	0.07	2.68	0.359
Exposure (high)	2.33	0.59	9.20	0.226	3.83	0.83	17.77	0.086
Exposure (medium)	2.50	0.73	8.61	0.146	3.20	0.82	12.42	0.093
Group (Ivermectin)	0.33	0.13	0.83	0.019	0.26	0.10	0.71	0.008

Kaplan-Meier risk analysis showed that on the seventh day there were seven patients with a COVID-19 infection in the control group and three patients in the ivermectin group; during the second week there were six patients with a COVID-19 infection in the control group and one patient in the ivermectin group. In the third week there were four patients with a COVID-19 infection in the control group and one patient in the ivermectin group. Finally, in the fourth week there was one patient with a COVID-19 infection in the control group and 0 patients in the ivermectin group.

**Figure 2:**
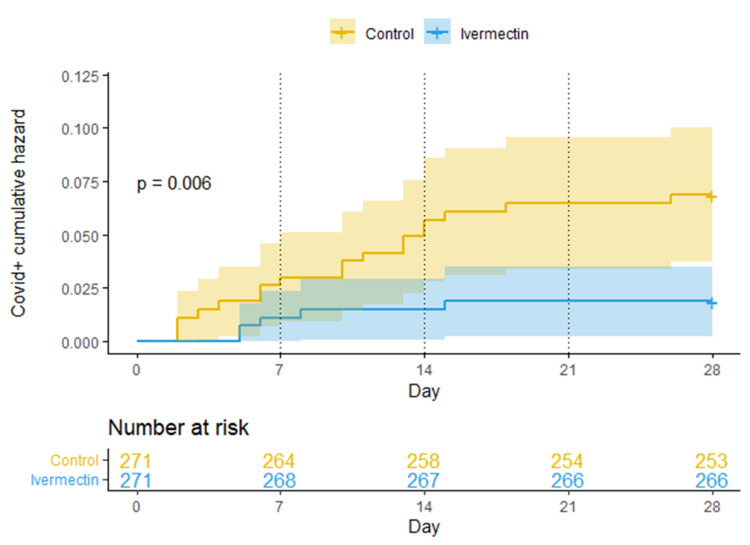
Kaplan-Meier cumulative risk curves for getting COVID-19 infection over the 28 days of this study. Log-rank method for p-value (95% CI).

The first four days after starting the study, a slightly higher number of positive COVID-19 infections was seen in the control group compared to the ivermectin group. Then, from the fifth day, the number of positive COVID-19 infections was the same in both groups. At day 10, a difference between the two groups began to be noticed, which increased over the course of the days until reaching day 28, where the protection of ivermectin achieved significant statistics (p-value = 0.006). Between days 16 and 28 (12 days), the ivermectin group did not present any contagion (Figure 2).

Post hoc analysis

**Figure 3:**
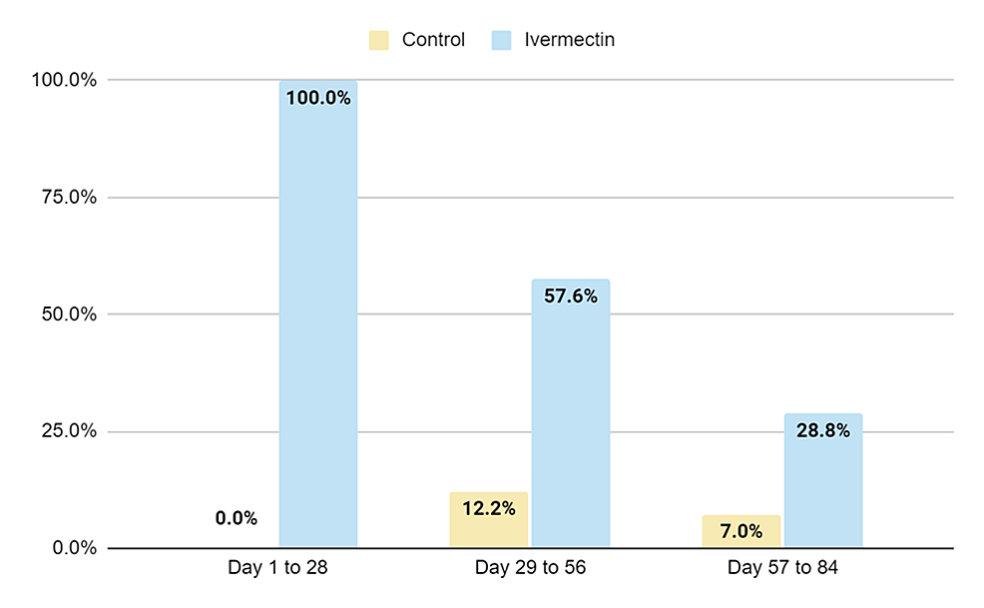
Ivermectin prophylactic program compliance for matched groups. The original 4 weeks with a weekly dose of Ivermectin, followed by 8 weeks with a monthly dose of Ivermectin.

**Figure 4:**
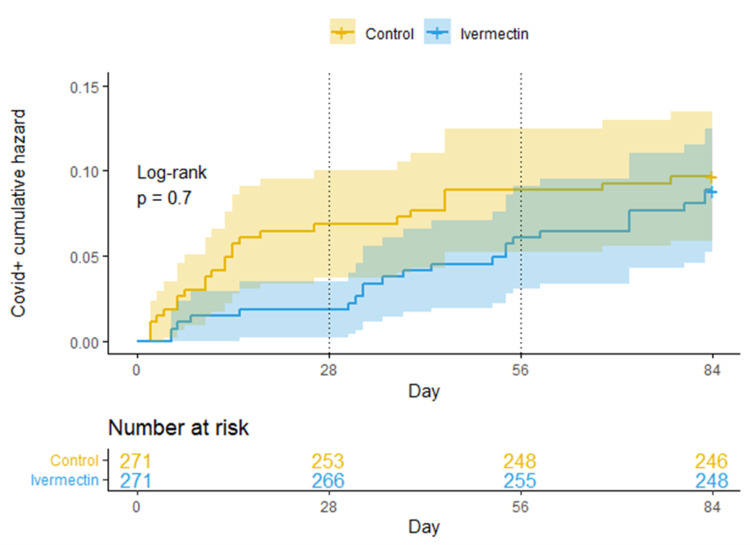
Kaplan-Meier cumulative risk curves for getting COVID-19 infection, extended version of Figure 2. The original 4 weeks of Ivermectin with a weekly dose and thereafter administering Ivermectin a monthly dose, during 8 weeks.

An additional eight-week follow-up was performed on the original matched groups (ivermectin and control), which passed from the weekly dose to a monthly dose PO 0.4 mg/kg. During these 56 days, most of the healthcare workers progressively discontinued ivermectin of their own will, for nonspecific reasons (Figure 3). When the ivermectin group was compared to the control group, there was no statistically significant difference regarding new cases of COVID-19 infections (Figure 4).

## Discussion

Ivermectin has a 19-hour plasma half-life, an enterohepatic cycle and its active metabolites can remain in the blood for up to 72 hours. Its excretion is 99% hepatic and only 1% is excreted in the urine. If we give successive doses of ivermectin on days one, four and seven, the drug accumulation in blood is minimal [8-9]. Ivermectin is very lipophilic and its concentration in some tissues is high; active levels in the lungs reach almost three times higher than in the blood and are detectable in fat tissue for at least seven days [10].

Kaplan-Meier risk analysis showed that the prevention with ivermectin occurred after the second dose was received on day eight. This finding could suggest that to achieve a preventive dose of ivermectin in the tissues, a second dose is needed (Figure 2).

This is consistent with the study results conducted in India by Behera and colleagues, which evaluated 91 healthcare workers exposed to patients with COVID-19 who received preventive ivermectin at 0.3 mg/kg on days one and four and were followed for one month. They compared this group with another group of 67 health workers that were not administered ivermectin. A 73% decrease risk in COVID-19 infection was observed in the ivermectin group versus the placebo group (OR = 0.26, 95% CI [0.14, 0.47]). COVID-19 infections in the group that received the two doses of ivermectin were not severe in any case and there were no cases of death. They reported a third group of 17 people who only took ivermectin on day one, in whom the preventive effect was not observed [11].

Chahla and colleagues, in Tucuman, Argentina, conducted a 1:1 randomized controlled study in healthcare workers for four weeks with a two-week follow-up after completion of the study. They compared a group that was administered PrEP with ivermectin PO 12mg weekly plus Iota-Carrageenan 6 nasal sprays daily, versus a control group that did not receive any medication. In the “Ivermectin-Carrageenan” group, four out of the 117 participants (3.4%) were infected with COVID-19, and in the control group 25 out of the 117 participants (21.4%) were infected. The difference between the two groups was statistically significant (p-value <0.05). The odds ratio (OR) was 0.13, this value transformed into relative risk (RR) becomes 84% less risk of contagion of SARS-CoV-2 in the “Ivermectin-Carrageenan” group in comparison with the control group. In the ivermectin group four patients had a mild infection and in the control group, in 15 patients the infection was mild, in seven moderate and in three severe [12].

In our study, the hazard ratio (HR) of 0.26 was achieved in the ivermectin group, which translates into a 74% lower risk of contagion with SARS-CoV-2. This risk result is similar to Behera et al., but 10 points less than the Chahla et al. study [11-12]. This additional difference could be attributed to the use of Iota-Carrageenan.

In the present study, when comparing healthcare personnel who presented COVID-19 infection, the progression of the disease, the appearance of complications, the need for hospitalization and the occurrence of death, the difference between both groups was not statistically significant (Table 2). These findings could be due to: 1. their average young age of 35 years old, where patients tend to have fewer complications with COVID-19 infections; 2. the study could be underpowered for the secondary outcomes; 3. the patients of both groups were offered early treatment with ivermectin, which diminishes the progression of SARS-CoV-2 infection, as seen in previous studies [6].

Since this is not a randomized study and the selection of groups was based on adherence, it does not allow us to clear certain confounding factors, such as the fact that the ivermectin group could be made up of healthcare personnel more concerned with prevention in general, including greater personal protection measures and more careful use of the PPE, reducing the risks of contracting SARS-CoV-2. To analyze this argument, we followed up the same participants of the ivermectin group (Figure 4) that passed from the weekly dose to a monthly dose PO 0.4 mg/kg and were followed for 56 days (eight weeks). During these 56 days, most of the healthcare workers progressively discontinued ivermectin of their own will, for nonspecific reasons (Figure 3). When the ivermectin group was compared to the control group, there was no statistically significant difference regarding new cases of COVID-19 infections. This finding makes the first argument unlikely.

Another element that may raise doubts could be the possibility that some participants in the control group used ivermectin on their own or another preventive drug. We consider that this probability is very low, since the hospital management widely provided the medicine to the health personnel of both medical centers and free of charge.

Limitations

The study was limited to 28 days, as in August 2020 the prevention scheme changed to a monthly dose of ivermectin 0.4 mg/kg. There was no RT-PCR test at the start or exit of this study, neither in the ivermectin group nor in the control group. There is the possibility that asymptomatic cases were not detected. Additionally, selection of groups was based on adherence.

## Conclusions

Pre-exposure prophylaxis (PrEP) for COVID-19 with a weekly oral dose of ivermectin 0.2 mg/kg was statistically significant in exposed healthcare personnel at the CMBO and CMPC after 28 days of follow up, with only 1.8% of the physicians and health collaborators developing SARS-CoV-2 infection versus 6.6% in the control group (p-value = 0.006). Ivermectin reduced the risk of contagion with COVID-19 by 74% compared to the control group (HR 0.26, 95% CI [0.10, 0.71]). These results suggest that compassionate use of weekly ivermectin could be an option as a preventive method in healthcare workers and as an adjunct to immunizations, while further well-designed randomized controlled trials are developed to facilitate scientific consensus.
